# Evolution of HER2-low expression from primary to recurrent breast cancer

**DOI:** 10.1038/s41523-021-00343-4

**Published:** 2021-10-12

**Authors:** Federica Miglietta, Gaia Griguolo, Michele Bottosso, Tommaso Giarratano, Marcello Lo Mele, Matteo Fassan, Matilde Cacciatore, Elisa Genovesi, Debora De Bartolo, Grazia Vernaci, Ottavia Amato, PierFranco Conte, Valentina Guarneri, Maria Vittoria Dieci

**Affiliations:** 1 Department of Surgery, Oncology and Gastroenterology (DISCOG), University of Padova, Padova, Italy; 2 Medical Oncology 2, Istituto Oncologico Veneto IOV-IRCCS, Padova, Italy; 3 Surgical Pathology Unit, University Hospital of Padua, Padua, Italy; 4 Department of Medicine (DIMED), Surgical Pathology & Cytopathology Unit, University of Padua, Padua, Italy; 5 Veneto Institute of Oncology IOV – IRCCS, Padua, Italy; 6 Department of Pathology and Molecular Genetics, Treviso General Hospital, Treviso, Italy

**Keywords:** Metastasis, Breast cancer, Tumour biomarkers

## Abstract

About a half of HER2-negative breast cancer (BC) show HER2-low expression that can be targeted by new antibody-drug conjugates. The main aim of this study is to describe the evolution of HER2 expression from primary BC to relapse by including HER2-low category in both primary and recurrent BC samples. Patients with matched primary and relapse BC samples were included. HER2 was evaluated according to ASCO/CAP recommendations in place at the time of diagnosis. A cutoff of >10% cells staining for HER2-positivity was applied. HER2-negative cases were sub-classified as HER2-low (IHC = 1 + /2+ and ISH not amplified), or HER2-0 (IHC-0). 547 patients were included. The proportion of HER2-low cases was 34.2% on the primary tumor and 37.3% on the relapse samples. Among HER2-negative cases, HER2-low status was more frequent in HR-positive vs triple-negative tumors (47.3% vs 35.4% on primary tumor samples, 53.8% vs 36.2% on relapse samples). The overall rate of HER2 discordance was 38.0%, mostly represented by HER2-0 switching to HER2-low (15%) and HER2-low switching to HER2-0 (14%). Among patients with a primary HER2-negative tumor, the rate of HER2 discordance was higher in HR-positive/HER2-negative vs triple-negative cases (45.5% vs 36.7% *p* = 0.170). This difference was mostly driven by cases switching from HER2-0 to HER2-low. HER2-low expression is highly unstable during disease evolution. Relapse biopsy in case of a primary HER2-0 tumor may open new therapeutic opportunities in a relevant proportion of patients.

## Introduction

Breast cancer is the most frequently diagnosed malignancy in women worldwide^1^. It represents a highly heterogeneous disease, encompassing diverse biological entities differing in terms of clinicopathologic features, prognosis and sensitivity to treatments. Although gene expression studies^2–6^ have revealed an impressive and complex biological diversity among breast tumors, in clinical practice breast cancer classification driving the treatment-decision process is based on the distinction between 3 major breast cancer subsets^2,7,8^, based on conventional immunohistochemistry (IHC) and in situ hybridization (ISH) analyses: hormone receptor (HR)-positive/HER2-negative, HER2-positive (HR positive or negative), triple-negative. The biological differences among these subtypes account for different clinical behaviors and distinct treatment sensitivity.

Although in common clinical practice the treatment-decision process in terms of access to anti-HER2 targeted agents is driven by the dichotomization in HER2-positive (as defined by HER2 protein overexpression on IHC analysis—score 3+ and/or *HER2* gene amplification on ISH assay) vs negative–which has been strengthened by 2018 ASCO/CAP guidelines^9^—HER2-negative breast cancer is characterized by a wide spectrum of HER2 expression levels^10^. Notably, preliminary results from early phase clinical trials testing novel anti-HER2 antibody-drug conjugates (ADCs) in advanced breast cancer patients harboring HER2-low expression (1 + or 2+ by IHC in the absence of *HER2* gene amplification by ISH) are challenging this notion^11,12^. In particular, in a phase Ib study of Trastuzumab Deruxtecan a notable 37% of ORR and 10.4 months of median duration of response were reported in HER2-low advanced breast cancer patients^12^. Similarly, in a phase Ib study investigating Trastuzumab Duocarmazine in patients with advanced solid tumors, ORR was 28% and 40% in the HR + HER2-low and triple-negative HER2-low breast cancer cohorts, respectively^11^. In addition, a large phase III trial investigating Trastuzumab Deruxtecan in pre-treated HER2-low advanced breast cancer patients is currently ongoing (NCT03734029/Destiny-Breast04).

Overall considered, HER2-low breast cancer is emerging as a distinct entity among the heterogenous population of HER2-negative tumors, and the proportion of breast cancer patients exhibiting HER2-low expression is not negligible, accounting for more than a half of all HER2-negative breast cancers^13,14^. However, a thorough evaluation of HER2-low evolution from primary to recurrent breast cancer is still lacking. In particular, it has been consistently demonstrated the phenomenon of both HR and HER2 status discordance between primary and recurrent breast cancer^15–17^, and biopsy of sites of locoregional relapse/distant metastasis is currently endorsed by international breast cancer guidelines^8,18,19^. Focusing on HER2 status, HER2 discordance has so far been investigated by dichotomizing cases between HER2-positive vs HER2-negative or, as recently reported, by considering the evolution from primary to metastatic disease of IHC-based scores in HER2-low primary breast cancer^20^. However, no evidence has so far been produced regarding the evolution of HER2 status by including HER2-low category in both primary and recurrent breast cancer samples.

The main aim of the present study is to evaluate the evolution of HER2-low expression from primary breast cancer to matched locoregional recurrences/distant metastases.

## Results

### Patient cohorts and clinicopathologic features

**Fig. 1:**
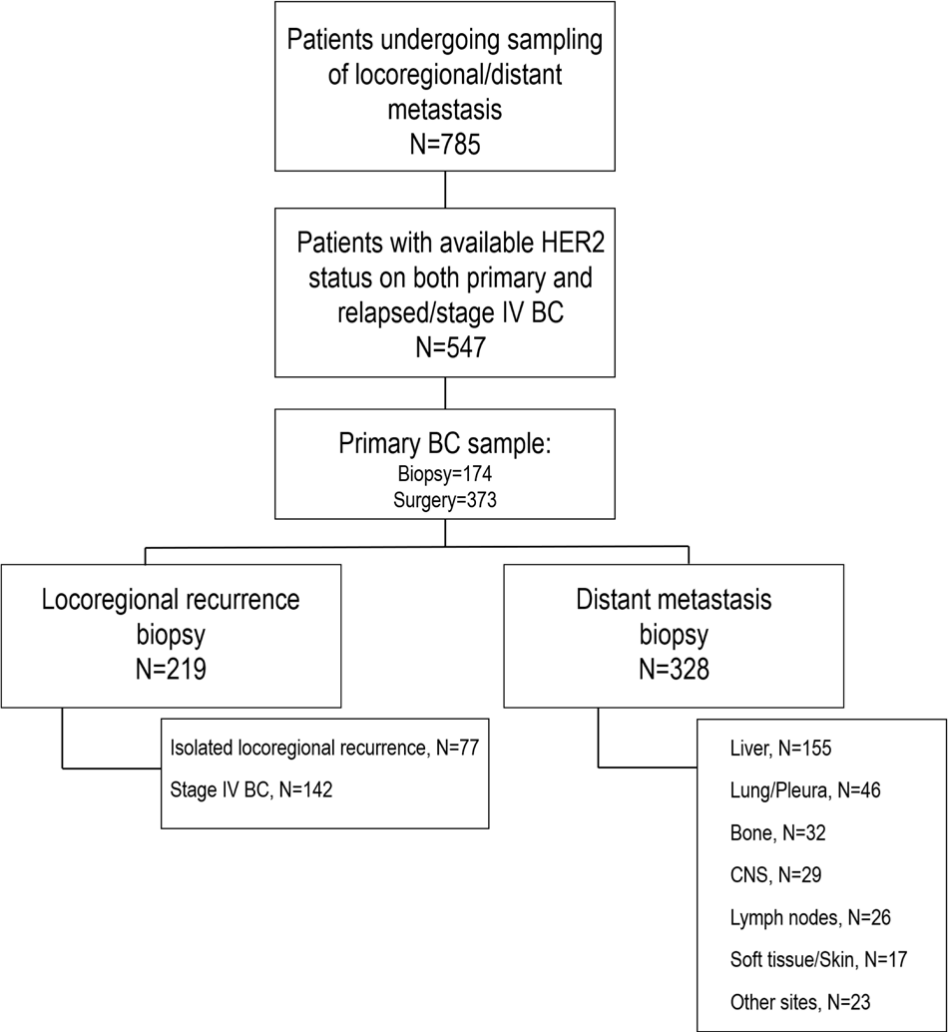
Flow diagram of the study. N number, BC breast cancer, CNS central nervous system.

A total of 547 patients who underwent tissue confirmation of locoregional recurrence or distant metastasis were included, as summarized in Fig. 1. 328 (60.0%) patients underwent biopsy of distant metastases, while 219 (40.0%) underwent sampling of locoregional sites, of whom 77 (14.0% of the total cases) experienced isolated locoregional recurrence. Main clinicopathological features at diagnosis of the overall cohort are summarized in Table 1.

**Table 1:** Clinicopathological features of the overall population at diagnosis.

	*N* (%) or median (Q1–Q3)
Total	547 (100)
Age at primary BC diagnosis (yy), median (Q1–Q3)	51.6 (43.4–62.6)
Primary BC histotype	
Ductal	437 (79.9)
Non-ductal	104 (19.0)
Missing	6 (1.1)
Primary BC histologic grade	
1	27 (5.0)
2	187 (34.2)
3	308 (56.3)
Missing	25 (4.5)
Stage at BC diagnosis	
Early	509 (93.1)
Stage IV	32 (5.9)
Missing	6 (1.1)
Primary BC phenotype	
HR + /HER2−	336 (61.4)
Triple-negative	79 (14.5)
HER2 +	132 (24.1)
Missing	0 (0.0)
Treatment for early BC	
Adjuvant	
Chemotherapy	282 (51.5)
Hormonal therapy	350 (64.0)
Anti-HER2	68 (12.4)
Neoadjuvant	
Chemotheapy	151 (27.6)
Hormonal therapy	14 (2.5)
Anti-HER2	37 (6.8)
Treatment for relapsed/stage IV BC	
Chemotherapy	430 (78.6)
Hormonal therapy	364 (66.5)
Anti-HER2	40 (7.3)
	131 (23.9)
Time from primary BC diagnosis and BC relapse (mo), median (Q1-Q3)	39.4 (20.3–81.5)
Time from recurrent BC diagnosis and biopsy of relapse (mo), median (Q1-Q3)	0.2 (0.0–6.9)

*BC* breast cancer, *yy* years, *Q* quartile, HR + /HER2 + , hormone-receptor positive/HER2-negative, mo months

Median time (Q1-Q3) between diagnosis of recurrent/stage IV breast cancer and tissue confirmation of locoregional recurrence/distant metastasis was 0.2 months (0.0–6.9).

The distribution according to the primary tumor phenotypes was as follows: HR-positive/HER2-negative 61.4% (*n* = 336), HR-negative/HER2-negative (triple-negative) 14.5% (*n* = 79), HER2-positive 24.1% (*n* = 132).

The distribution according to the recurrent tumor phenotypes was as follows: HR-positive/HER2-negative 55.0% (*n* = 301), triple-negative 21.2% (*n* = 116), HER2-positive 23.4% (*n* = 128).

### Features of HER2-low phenotype in primary and recurrent/stage IV breast cancer

In the overall cohort (*N* = 547), the proportion of HER2-low cases was 34.2% (*n* = 187) of primary breast cancer samples and 37.3% (*n* = 204) of recurrent/stage IV samples, accounting for the 45.0% and the 48.9% of the entire HER2-negative primary breast cancer and recurrent cohorts, respectively.

Among HER2-negative cases, HER2-low distribution according to breast cancer subtype is summarized in Table 2. In particular, a positive association was observed between HER2-low expression and HR-positive/HER2-negative breast cancer subtype in both primary and recurrent/stage IV breast cancer. In particular, HER2-low expression represented 47.3% of primary HR-positive/HER2-negative vs 35.4% of triple-negative cohort, respectively (*p* = 0.060) and 53.8% vs 36.2% of recurrent/stage IV HR-positive/HER2-negative vs triple-negative cohort, respectively (*p* = 0.001). The distribution of HER2 expression by IHC according to tumor phenotype in the HER2-low primary and recurrent BC cohort is summarized in Table 3.

**Table 2:** HER2 expression distribution according to breast cancer subtype in the HER2-negative primary and recurrent breast cancer cohort.

	HER2 expression *n* (%)		
	0	Low	*p*
Primary breast cancer *n* (%)			
HR-positive/HER2-negative	177 (52.7)	159 (47.3)	0.060
Triple-negative	51 (64.6)	28 (35.4)	
Recurrent breast cancer *n* (%)			
HR-positive/HER2-negative	139 (46.2)	162 (53.8)	0.001
Triple-negative	74 (63.8)	42 (36.2)

**Table 3:** Distribution of HER2 expression by IHC according to tumor phenotype in the HER2-low cohort.

	HER2 expression *n* (%)		
	1+	2+	*p*
Primary breast cancer *n* (%)			
HR-positive/HER2-negative	118 (63.1)	41 (21.9)	0.493
Triple-negative	19 (10.2)	9 (4.8)	
Total	137 (73.3)	50 (26.7)	
Recurrent breast cancer *n* (%)			
HR-positive/HER2-negative	122 (59.8)	40 (19.6)	0.840
Triple-negative	33 (16.2)	9 (4.4)	
Total	155 (76.0)	49 (24.0)	

Main clinicopathological features of HER2-negative tumors according to HER2-low expression and tumor phenotype in primary and recurrent breast cancer cohorts are summarized in Supplementary Tables 1 and 2, respectively.

HER2 expression according to different types of tumor samples is reported in Supplementary Table 3. In primary BC samples, HER2-low expression was more represented in biopsies as compared to surgical specimens (54.6% vs 41.2%, *p* = 0.016). In BC recurrences, similar rates of HER2-low cases were observed between locoregional relapses and distant metastases and across different metastatic sites. In HR-positive/HER2-negative primary breast cancer cohort, a statistically significant association was observed between HER2-low expression and both Luminal B-like phenotype (Luminal B-like proportion in HER2-0 vs HER2-low: 51.9% vs 64.7%, respectively, *p* = 0.023) and higher ER expression (mean ER expression in HER2-0 vs HER2-low: 71.2% vs 76.8%, *p* = 0.032).

In HR-positive/HER2-negative recurrent breast cancer cohort, HER2-low cases were associated with younger age at primary breast cancer diagnosis as compared to HER2-0 cases (*p* = 0.018).

### HER2 evolution from primary breast cancer to recurrent/stage IV breast cancer

**Fig. 2:**
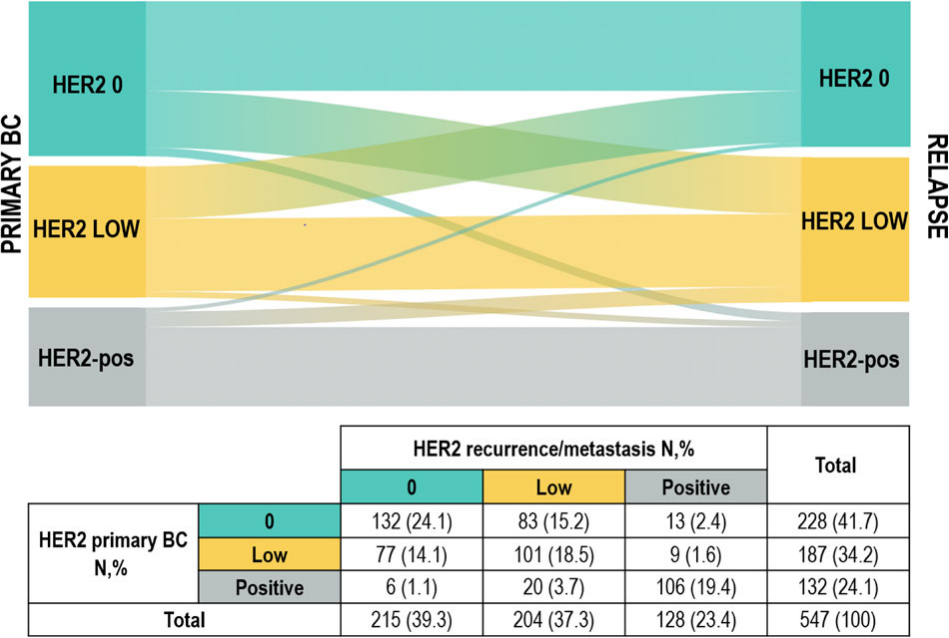
HER2 expression evolution from primary BC to relapse. Figure 2 shows the evolution of HER2 expression from primary to recurrent breast cancer. Absolute numbers and percentages are reported. BC breast cancer, N number.

Figure 2 summarizes HER2 evolution from primary breast cancer to secondary lesions. The overall rate of HER2 discordance was 38.0% (*n* = 208), mostly driven by cases switching to or from HER2-low expression. In particular, in 15.2% of the cases a conversion from HER2-0 to HER2-low phenotype was observed (36.4% of the HER2-0 primary breast cancer cohort), and in 14.1% of patients a change from HER2-low primary breast cancer to HER2-0 recurrent/stage IV breast cancer was found (41.2% of the HER2-low primary breast cancer cohort). HER2-positive phenotype in either primary or recurrent breast cancer samples showed the highest stability, with 4.8% of the total cases exhibiting loss or gain of HER2 positivity. Among HER2-positive breast cancer patients showing HER2 loss from primary to recurrent breast cancer (*n* = 26), the great majority (*n* = 20, 77%) maintained some level of HER2 expression, exhibiting a HER2-low phenotype.

Main clinicopathologic features according to HER2 discordance status are shown in Supplementary Table 4.

HER2 evolution according to IHC scores is shown in Supplementary Fig. 1.

**Fig. 3:**
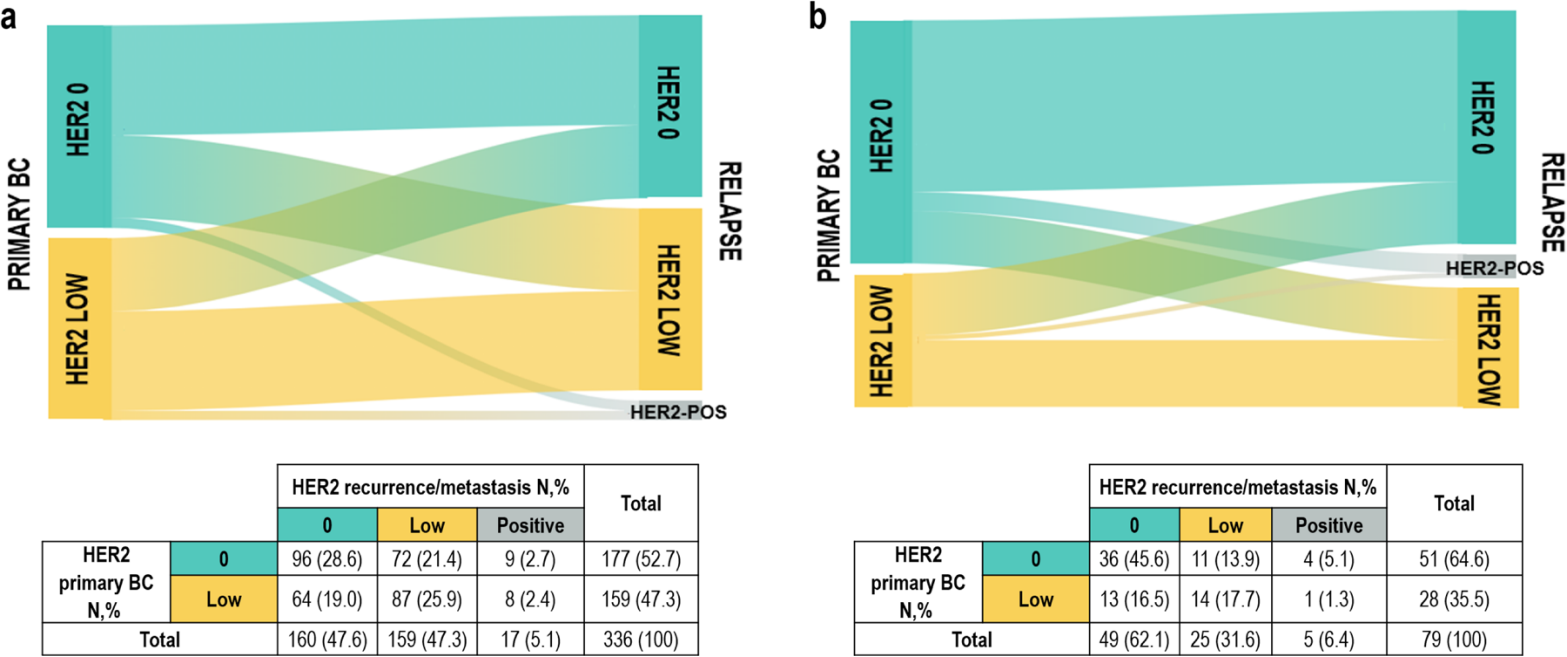
HER2 expression evolution from primary BC to relapse according to breast cancer phenotype. **a** HR-positive/HER2-negative phenotype. **b** triple-negative phenotype. BC breast cancer, N number.

Among patients with HER2-negative primary breast cancer, the proportion of HER2 discordant cases was numerically higher in HR-positive/HER2-negative cohort (HER2 discordant cases in HR-positive/HER2-negative vs triple-negative: 45.5% vs 36.7%, respectively, *p* = 0.17) and this difference was mainly driven by the proportion of HR-positive/HER2-0 breast cancer cases switching to HER2-low expression. In detail, as shown in Fig. 3A, among HR-positive/HER2-negative cases, 21.4% switched from HER2 0 to HER2-low expression, while 19.0% converted from HER2-low to HER2 0 breast cancer. Among triple-negative primary breast cancer, in 13.9% of the patients a conversion from HER2-0 to HER2-low phenotype was observed, while in 16.5% a conversion in the opposite direction occurred (Fig. 3B).

Table 4 dissects HER2 expression conversion in HR-positive/HER2-negative and triple-negative cohorts according to the tumor phenotype of the secondary lesion. In detail, 37.2% of HR-positive/HER2-negative patients and 25.6% of triple-negative patients showed a conversion from or to HER2-low expression while maintaining the same breast cancer phenotype.

**Table 4:** HER2 expression evolution from HER2-negative primary breast cancer to HER2-negative recurrent breast cancer according to breast cancer phenotype.

	HER2 expression on recurrence/metastasis				
	HR + /HER2−		Triple-negative		Total
	0	Low	0	Low	
HER2 expression on primary breast cancer					
HR + /HER2−					
0	74 (18.9)	64 (16.4)	21 (5.4)	8 (2.0)	167 (42.7)
Low	54 (13.9)	78 (20.0)	9 (2.3)	9 (2.3)	150 (38.5)
Triple-negative					
0	5 (1.2)	3 (0.8)	31 (7.9)	8 (2.0)	47 (11.9)
Low	2 (0.5)	3 (0.8)	11 (2.8)	11 (2.8)	27 (6.9)
Total	135 (34.5)	148 (38.0)	72 (18.4)	36 (9.1)	391 (100)

*BC* breast cancer

Among HR + /HER2- and TN cases, no association between HER2 discordance and type of adjuvant treatments (endocrine therapy and/or chemotherapy, and chemotherapy, respectively) was observed (data not shown).

### Evolution of HER2 expression according to tumor sample

The overall rate of HER2 discordance did not differ according to type of primary breast cancer sample. In particular, the overall rate of HER2 discordance was 37.5% vs 39.1% when primary BC phenotype was assessed on surgical samples and biopsies, respectively (*p* = 0.777).

Moreover, HER2 discordance rate was 36.1% vs 39.3% when relapsed BC phenotype was assessed on locoregional and distant sites, respectively (*p* = 0.473). Conversely, HER2 discordance rate was significantly different across different metastatic sites (*p* = 0.001), with higher rates observed for liver (49.7%) and bone (43.8%), followed by soft tissue/skin (39.8%), lymph-node (36.3%), other sites (30.4%), lung/pleura (21.7%), and central nervous system (CNS,13.8%). The direction of discordance in liver and bone was equally represented by losses and gains of HER2 expression.

### Exploratory survival analysis

For exploratory survival analyses, we focused on the subgroup of patients exhibiting HR-positive/HER2-negative BC phenotype in both primary and recurrent BC (*n* = 270). No significant PRS differences were observed according to four categories defining HER2 evolution (HER2-0/HER2-0; HER2-0/HER2-low; HER2-low/HER2-0 and HER2-low/HER2-low, *p* = 0.063). Similar findings were observed by excluding patients receiving CDK 4/6 inhibitors in the advanced setting (*n* = 33), by adopting May, 2017 as cutoff (when CDK 4/6 inhibitors were made available in our region). When focusing on HER2 expression on primary tumor, a significant association between HER2 expression and survival was observed. In particular, among patients preserving HR-positive/HER2-negative BC subtype during disease evolution, those with HER2-low expression on primary tumor experienced significantly poorer PRS and OS as compared to patients with HER2-0 primary BC (HER2-0 vs HER2-low: median PRS 53.2 vs 41.7 months, respectively, hazard ratio 0.71 (95% CI 0.53-0.96), *p* = 0.025; median OS 128.2 vs 100.3 months, respectively, hazard ratio 0.72 (95% CI 0.54–0.97), *p* = 0.030). Kaplan Meier curves are shown in Supplementary Fig. 2A–C.

## Discussion

HER2-low breast cancer is emerging as a novel entity, thus contributing to the biological and clinical complexity of breast cancer. In the present work we evaluated in a large cohort the evolution of HER2-low expression from primary to recurrent breast cancer by including the HER2-low category in the characterization of both primary and secondary breast cancer samples.

In our large cohort of 547 breast cancer patients with paired samples of primary tumor and locoregional/distant metastases, HER2-low breast cancer accounted for almost a half of the entire HER2-negative cohort (45%), consistently with available evidence^21^. In addition, we observed a numerically higher proportion of HER2-low cases among recurrent breast cancer samples than primary tumors, similarly to a previous retrospective study in which HER2-low cases enriched the metastatic cohort as compared to the primary breast cancer population^19^.

Interestingly, we found an association between HR status and HER2-low expression. In particular, HR-positive/HER2-negative breast cancer cohort was enriched for HER2-low tumors patients in both primary breast cancer and recurrent/metastatic lesions, with HER2-0 tumors instead enriching the triple-negative cohort. This observation appears consistent with previous studies reporting higher prevalence of HR-positive/HER2-negative rather than triple-negative phenotype among HER2-low breast tumors^13,22–26^. We also found higher ER levels in the HER2-low as compared to HER2-0 cohort in our HR-positive/HER2-negative subgroup. These findings overall strengthen the hypothesis that the complex crosstalk between HER2 and HR pathways may play a crucial role in biologically defining the HER2-low phenotype^20^. Of course, this observation needs to be further verified in future studies. We also observed an association between Luminal B-like subtype and HER2-low expression in HR-positive/HER2-negative breast cancer patients. This finding appears in contrast with a recent large retrospective study in which an association between HER2-low phenotype and both lower expression of proliferative genes and Luminal-A PAM50-based intrinsic subtype was reported^13^. However, it should be noted that in our study the definition of Luminal B-like phenotype relied on an IHC-based assessment of Ki67, thus complicating the comparability of our results with those obtained by gene-expression analyses. Indeed, in the above-mentioned study, no association between HER2-low phenotype with Ki67 IHC-based scores was observed. Interestingly, among HER2-negative cases, HER2 expression on recurrence was not affected by the type of sample, either when comparing locoregional relapses vs distant metastases or across different metastatic sites. The lack of significant variability across different anatomical sites represents an important finding, especially in view of the possible introduction of anti-HER2 agents for HER2-low patients in the future, since it reassures on the reliability of relapse BC phenotype irrespectively from the choice of the anatomical site to be subjected to biopsy. On the other hand, when comparing HER2 expression according to the type of primary tumor sample, higher rates of HER2-low cases were observed when the primary tumor phenotype was assessed on biopsy as compared to surgical samples. Since in our study all primary tumor samples were treatment-naïve, an actual biological diversity could be reasonably excluded. It is rather conceivable to hypothesize a possible role for both intra-tumoral heterogeneity and pre-analytical factors (such as cold ischemia time differences). Indeed, although it is currently believed that core-needle-biopsy represents an acceptable sample for HER2 expression assessment^9^, so far this consideration has been drawn by focusing on the dichotomy between HER2-positive versus HER2-negative cases. However, with the potential inclusion of the HER2-low category among breast cancer predictive factors, future efforts should be addressed on determining the reliability of information obtained from core-needle-biopsy with respect to the surgical specimen in terms of both specificity and sensitivity.

Our main aim was to investigate the evolution of HER2-low expression from primary breast cancer to matched samples of locoregional recurrences or distant metastases. In the overall cohort, HER2 discordance rate was 38%, mostly driven by cases switching to or from HER2-low expression. In particular, more than one third of HER2-0 primary breast cancer patients exhibited a conversion towards HER2-low expression in the metastatic lesion, and more than 40% of patients with HER2-low expression in the primary tumor switched towards HER2-0 recurrent/stage IV breast cancer. A previous small unpublished study described that 47% of HER2-0 primary breast cancer showed an increase in the HER2 IHC score on relapse biopsy and that 40% of HER2-low primary tumors showed a decrease in the HER2 IHC score, confirming the instability of low HER2 expression^20^. However, authors stratified HER2 expression modifications between primary and recurrent breast cancer according to equal/increase/decreased IHC scores, thus precluding any specific comparison with our results.

The great instability of HER2-low expression either from or to HER2-0 phenotype suggests that that retesting HER2 expression on secondary lesions may be of worth, also in the light of the promising activity of novel anti-HER2 agents in HER2-low metastatic breast cancer^11,12^. It is also worth mentioning that HER2-low expression discordance either from or to HER2-0 phenotype was mainly driven by the HR-positive/HER2-negative subgroup, which showed numerically higher conversion rates as compared to the triple-negative subpopulation, especially when considering HR-positive/HER2-0 cases switching to HER2-low phenotype. This observation may deserve to be accounted when dealing with HR-positive/HER2-negative advanced breast cancer patients exhausting the main treatment options, including hormonal strategies and chemotherapy, but who may still benefit from additional treatments. In this context, those exhibiting HER2-low expression might represent ideal candidates for the inclusion in ongoing clinical trials of anti-HER2 ADCs and, more importantly, in case these emerging strategies were to become available for HER2-low BC in the near future. Conversely, although HER2-low evolution was less frequently observed in our triple-negative cohort than the HR-positive one, almost 30% of triple-negative breast cancer patients exhibited a conversion either from or to HER2-low expression. This observation may be of special value in the challenging scenario of triple-negative advanced breast cancer, where the possibility to open new therapeutic options appears particularly appealing, even in the earliest phases of advanced disease.

It should additionally be noted that a not negligible proportion of HR-positive/HER2-negative (~35%) and triple-negative (~25%) cases switched towards or from HER2-low expression while maintaining the same breast cancer phenotype during disease evolution. Of course, the possible inclusion of anti-HER2 ADCs in the treatment armamentarium for HER2-low breast cancer is still far from the implementation in the clinical practice, thus limiting the contextualization of our observation in a contemporary clinical scenario, also given the actual lack of solid evidence supporting the adoption of the metastatic breast cancer phenotype rather than that of the primary tumor in the treatment-decision process of advanced disease. However, our finding suggests that the evolution of HER2-low expression from primary to recurrent tumor within the same breast cancer subtype involves a not negligible proportion of HER2-negative breast cancer patients, thus surely deserving to be further deepened. In our study, as expected, and consistently with available evidence^15,17^, HER2-positive phenotype in either primary tumors or recurrent lesions was the most stable, with less than 5% of the total cases showing HER2 positivity gain or loss.

Another worth noting observation is that, in our cohort, HER2 discordance appeared to be affected by the type of relapse sample, with liver and bone metastases being associated with the highest discordance rates and lung and CNS metastases instead showing the lowest rates of HER2 discordance, as compared to other metastatic sites. Since, as already discussed, HER2 expression did not differ across different anatomical locations, this finding may imply an actual biological background or, rather, a (pre)-analytical explanation, especially when considering the high discordance observed in bone lesions. In this regard, although in our study we only included bone metastases considered reliable by the pathologist, a possible role of the decalcification process cannot be excluded in this regard. However, the direction of HER2 discordance in bone lesions was not enriched for HER2 expression losses, thus downsizing the impact of this possible bias. Although the absence of literature data in this regard precludes the possibility to draw definitive conclusions, a possible pragmatical implication of these findings, if confirmed in future studies, is the possibility to strategically select the site of metastasis to be biopsied in order to maximize the opportunity of access to novel anti-HER2 drugs for patients originally classified as HER2-0 based on the primary tumor phenotype.

We also performed an exploratory analysis aiming at assessing possible survival differences according to HER2-low expression evolution from primary to recurrent BC. We decided to focus on the subgroup of patients maintaining HR-positive/HER2-negative BC phenotype during disease evolution given the high HER2-low expression instability observed in this subgroup. No significant PRS impact of either HER2-low concordance or discordance was observed. We hypothesized that the major determinant for PRS in this cohort would be HER2 expression on primary BC. Indeed, a significantly decrease in both PRS and OS was observed in patients with HR-positive/HER2-low primary breast cancer as compared to those with HR-positive/HER2-0 phenotype. A possible explanation for such observation is that our HR-positive/HER2-low primary BC cohort was enriched for cases with Luminal B-like phenotype, which is known to independently correlate with poorer outcome as compared to Luminal A-like breast cancer^27^. Although results from this survival analysis should be interpreted with caution given the mere exploratory nature, they appear consistent with available scattered retrospective data supporting a possible negative prognostic impact of HER2-low expression in early breast cancer patients^24,27–29^, especially when considering the HR-positive subset^28^. However, a prognostic association in the opposite direction has been reported as well^30,31^, thus precluding the possibility to draw definitive conclusions in this regard. In addition, it should be noted that in the abovementioned studies heterogeneous definitions for the identification of HER2-low expressing tumors were adopted, thus further complicating the interpretation of results. Indeed, additional efforts should be made in order to deepen the possible impact of this emerging biomarker on survival of breast cancer patients with advanced disease.

Our findings overall stress the importance to retest HER2 when dealing with recurrent and/or stage IV breast cancer patients. Indeed, as already mentioned, the identification of HER2-low expression on locoregional recurrences or distant metastases in patients with originally HER2-0 primary breast cancer may expand patients’ therapeutic scenario by offering the opportunity to access ongoing clinical trials testing anti-HER2 ADCs in this breast cancer subgroup. However, it is currently largely unknown whether HER2-low breast cancer patients exhibiting a complete loss of HER2 expression during disease evolution may still benefit from these novel treatment strategies. It should be noted that these emerging anti-HER2 ADCs were investigated on HER2-low patients defined by HER2 testing in archival tissue of the last available biopsy, not necessarily corresponding to secondary lesions, thus precluding the possibility to draw conclusion in this regard.

One of the major strengths of the present work is that this represents one of the largest breast cancer study investigating not only the clinical features of patients harboring HER2-low expression, but also the evolution of this biomarker throughout the natural history of the disease. In addition, we adopted the HER2-low as a distinct category for the characterization of both primary and matched recurrent tumor samples. Furthermore, its multicentric nature allowed to minimize the biases related to a single-laboratory HER2 evaluation. Moreover, although HER2 expression was locally evaluated according to ASCO/CAP recommendations in place at the time of diagnosis, it should be noted that a 10% cut-off of cells staining for HER2 positivity was applied. For this reason, all cases diagnosed between 2007 and 2013 were centrally reviewed to comply with the 10% cut-off (in the ASCO/CAP recommendations in place in the period 2003-2013, the % of cells staining for HER2 required for the definition of IHC score 3+ was raised to 30).

Some limitations need to be highlighted as well. Firstly, the retrospective nature of the present study may have been responsible for the enrichment of our cohort for patients with unusual disease behaviors. In addition, a possible role of systemic treatments for advanced disease in affecting HER2 expression on secondary lesions cannot be excluded. However, it should be noted that the median time from diagnosis of relapse/stage IV breast cancer and biopsy of locoregional/distant metastases was 0.2 months, thus downsizing the contribution of this possible confounding factor.

In addition, a comprehensive central revision for HER2 expression was not performed in our study. However, 100 random samples were centrally and blinded reviewed for HER2 expression, showing a good overall agreement with the local assessment.

It should also be noted that no formal definition is currently available for HER2-low expression, and its identification is intrinsically dependent on the methodology applied to test HER2 levels. In the present study we applied IHC and ISH to define HER2-low tumors, which represent the only standardized and established procedures to assess HER2 status. Accordingly, HER2-low tumors were defined as HER2 1+ and 2+ scores by IHC in the absence of gene amplification by ISH, consistently with previous studies1^11–13^^,^^20,21,24,31^. However, the analytical reliability of both IHC and ISH techniques are suboptimal. In fact, HER2 IHC staining and scores interpretation may be affected, among others, by the type of HER2 antibody clones, formalin-fixations variables and artifacts, as well as intratumoral heterogeneity^32^. In addition, so far, IHC 0 and IHC 1+ have been frequently pooled under the broader definition of HER2-negative breast cancer, with HER2-0 prevalence widely ranging from 15% to 80% across studies. In this context, concerning disagreement rates of HER2-0 IHC-based scores between local and central assessment (~85%) have been reported^33^. In addition, in a large retrospective study investigating the reproducibility of HER2 IHC classification (0 vs 1+ vs 2+ vs 3 + ), although a substantial overall agreement was reported across 5 breast cancer-specialized pathologists (multi-rater overall kappa concordance Score=0.79), it should be noted that the discordancy was mostly driven by cases which were discordant between 0 vs 1 + (~43% of all discordant cases, 15% of total cases)^13^. With the emergence of novel treatment strategies specifically directed to patients with HER2-low tumors, a stricter adherence to FDA/ASCO-CAP rules and recommendations for IHC HER2 scoring would be advisable. In addition, given the possible sub-optimality of the IHC/ISH-based method in the detection of HER2-low breast cancer patients, alternative technologies for a quantitative evaluation of HER2 have been suggested as potentially capable of improving our ability to identify this emerging breast cancer subset^34–39^, however none of them have yet been formally validated.

In conclusion, we showed that HER2-low expression is highly unstable during disease evolution. In this context, relapse biopsy in case of a primary HER2-0 tumor may open new therapeutic opportunities in a not negligible proportion of breast cancer patients. Indeed, HER2-low expressing breast cancer is emerging as a novel distinct entity, possibly challenging the current diagnostic-therapeutic paradigm, shifting from a 2-tier to a 3-tier algorithm, encompassing HER2-“truly”-negative, HER2-positive and HER2-low tumors.

## Methods

### Population

Breast cancer patients undergoing biopsy or surgical resection of either locoregional recurrence or distant metastasis at two different Italian Institutions (Istituto Oncologico Veneto—IRCCS, Padova and Treviso Hospital, Italy) between January 1999 and December 2019 were identified. Those patients for whom HER2 status evaluation was available on both primary tumor and matched relapse samples were included in this analysis. Patients experiencing contralateral breast cancer in the absence of other sites of recurrence were excluded.

Tumor samples of primary breast cancer and paired locoregional recurrence/distant metastases were retrieved from the Archive of the respective Pathology Departments.

Patients clinicopathologic characteristics including age, stage at diagnosis, HR status and expression, HER2 status and expression, timing and site of locoregional and/or distant relapse, and survival status were recorded.

### Pathology

HER2 status was considered positive in case of score 3+ by IHC and/or HER2 amplification by ISH, while HER2 was classified as negative in case of score 0/1 + /2+ by IHC in the absence of HER2 amplification by ISH (when applicable). HER2 status was retrieved from the original pathology report and was evaluated according to ASCO/CAP recommendations in place at the time of diagnosis (Data regarding IHC protocols adopted for the evaluation of HER2 are detailed in supplementary material)^9,40,41^. However, all cases diagnosed between 2007 and 2013 were reviewed by IHC to comply with the 10% cut-off of cells staining for HER2-positivity (*N* = 291 primary BC samples and *N* = 188 relapsed samples). Moreover, in order to confirm the analytical reliability of the present work, 100 random samples of matched cases underwent blind revision for HER2 levels by one expert pathologist according to current ASCO/CAP recommendations^40^, showing an 80% agreement with the original report. For this work, HER2-negative cases were further subclassified as HER2 0 in case of IHC Score=0, and HER2-low in case of IHC Score=1 + /2+ in the absence of *HER2* gene amplification by ISH.

Both estrogen receptor (ER) and progesterone receptor (PgR) status were retrieved from the original pathology report and were classified as positive in case of positive IHC staining in at least 10% of cancer cells; HR status was considered positive in case of ER and/or PgR positivity, while HR status was classified as negative in case of negativity of both ER and PgR.

For the evaluation of primary BC, surgical samples, when available, were preferred over biopsies, with the exception of cases undergoing neoadjuvant treatment, for whom baseline biopsy was instead used.

### Statistical analysis

Statistical analyses were carried out using IBM SPSS Statistics (version 22.0), software (IBM Corp, Armonk, NY, USA).

Descriptive statistics were performed for patient demographics and clinical characteristics. For continuous variables mean, median, range values, and quartiles were computed. Student-T test, and The Mann-Whitney and Kolmogorov-Smirnov nonparametric tests were used to study the distribution of continuous variables across groups defined by clinicopathologic characteristics. Chi-squared test (χ2) was used to compare categorical variables across subgroups. Sankey diagrams were built to depict the evolution of HER2 expression from primary to recurrent breast cancer.

Post-recurrence survival (PRS) was defined as the time from the date of diagnosis of locoregional relapse/distant metastasis and the date of death or last follow-up. Overall survival was defined as the time from the date of primary breast cancer diagnosis and the date of death or last follow-up. The Kaplan-Meier method was adopted to estimate survival curves and the log-rank test was used to test for differences across groups. The Cox-regression model was adopted to calculate hazard ratios and 95% confidence intervals (CI).

All reported *p* values are two-sided, and significance level was set at *p* < 0.05.

### Ethical statement

In the present study tumor samples were collected after approval by the respective Institutional Review Boards and in accordance with the Declaration of Helsinki. All the patients provided written informed consent prior to inclusion into the study.

### Reporting summary

Further information on research design is available in the Nature Research Reporting Summary linked to this article.

## Supplementary information

Reporting Summary

Supplementary Information

## Data Availability

The datasets that support the findings of this study are not publicly available in order to protect patient privacy. The data will be available on reasonable request from the corresponding author: VG, valentina.guarneri@unipd.it.
