# Author Correction: Evolution of HER2-low expression from primary to recurrent breast cancer

**DOI:** 10.1038/s41523-021-00359-w

**Published:** 2021-11-24

**Authors:** Federica Miglietta, Gaia Griguolo, Michele Bottosso, Tommaso Giarratano, Marcello Lo Mele, Matteo Fassan, Matilde Cacciatore, Elisa Genovesi, Debora De Bartolo, Grazia Vernaci, Ottavia Amato, PierFranco Conte, Valentina Guarneri, Maria Vittoria Dieci

**Affiliations:** 1 Department of Surgery, Oncology and Gastroenterology (DISCOG), University of Padova, Padova, Italy; 2 Medical Oncology 2, Istituto Oncologico Veneto IOV-IRCCS, Padova, Italy; 3 Surgical Pathology Unit, University Hospital of Padua, Padua, Italy; 4 Department of Medicine (DIMED), Surgical Pathology & Cytopathology Unit, University of Padua, Padua, Italy; 5 Veneto Institute of Oncology IOV-IRCCS, Padua, Italy; 6 Present Address: Department of Pathology and Molecular Genetics, Treviso General Hospital, Treviso, Italy

**Keywords:** Metastasis, Breast cancer, Tumour biomarkers

Correction to: *npj Breast Cancer* 10.1038/s41523-021-00343-4, published online 12 October 2021

In this article, the affiliation ‘Veneto Institute of Oncology IOV – IRCCS, Padua, Italy’ for Matteo Fassan was missing. The original article has been corrected.

