# Supplementary material for: Evolution of HER2-low expression from primary to recurrent breast cancer
Source: NPJ Breast Cancer. 2021 Oct 12;7:137. doi: 10.1038/s41523-021-00343-4 (PMC8511010; doi:10.1038/s41523-021-00343-4)
Supplement: Supplementary file 1 — Reporting Summary [file 41523_2021_343_MOESM1_ESM.pdf]

nature portfolio

Corresponding author(s): Valentina Guarneri
Last updated by author(s): Jun 22, 2021

Reporting Summary

Nature Portfolio wishes to improve the reproducibility of the work that we publish. This form provides structure for consistency and transparency in reporting. For further information on Nature Portfolio policies, see our Editorial Policies and the Editorial Policy Checklist.

Statistics

For all statistical analyses, confirm that the following items are present in the figure legend, table legend, main text, or Methods section.

n/a	Confirmed	
☐	☒	The exact sample size (*n*) for each experimental group/condition, given as a discrete number and unit of measurement
☐	☒	A statement on whether measurements were taken from distinct samples or whether the same sample was measured repeatedly
☐	☒	The statistical test(s) used AND whether they are one- or two-sided<br>*Only common tests should be described solely by name; describe more complex techniques in the Methods section.*
☐	☒	A description of all covariates tested
☒	☐	A description of any assumptions or corrections, such as tests of normality and adjustment for multiple comparisons
☐	☒	A full description of the statistical parameters including central tendency (e.g. means) or other basic estimates (e.g. regression coefficient) AND variation (e.g. standard deviation) or associated estimates of uncertainty (e.g. confidence intervals)
☐	☒	For null hypothesis testing, the test statistic (e.g. *F*, *t*, *r*) with confidence intervals, effect sizes, degrees of freedom and *P* value noted<br>*Give P values as exact values whenever suitable.*
☒	☐	For Bayesian analysis, information on the choice of priors and Markov chain Monte Carlo settings
☒	☐	For hierarchical and complex designs, identification of the appropriate level for tests and full reporting of outcomes
☒	☐	Estimates of effect sizes (e.g. Cohen's *d*, Pearson's *r*), indicating how they were calculated

Our web collection on statistics for biologists contains articles on many of the points above.

Software and code

Policy information about availability of computer code

Data collection	N/A
Data analysis	Statistical analyses were carried out using IBM SPSS Statistics (version 22.0), software (IBM Corp, Armonk, NY, USA).

For manuscripts utilizing custom algorithms or software that are central to the research but not yet described in published literature, software must be made available to editors and reviewers. We strongly encourage code deposition in a community repository (e.g. GitHub). See the Nature Portfolio guidelines for submitting code & software for further information.

Data

Policy information about availability of data

All manuscripts must include a data availability statement. This statement should provide the following information, where applicable:

- Accession codes, unique identifiers, or web links for publicly available datasets
- A description of any restrictions on data availability
- For clinical datasets or third party data, please ensure that the statement adheres to our policy

The datasets that support the findings of this study are not publicly available in order to protect patient privacy.
The data will be available on reasonable request from the corresponding author: VG, valentina.guarneri@unipd.it.

Field-specific reporting

Please select the one below that is the best fit for your research. If you are not sure, read the appropriate sections before making your selection.

☒ Life sciences ☐ Behavioural & social sciences ☐ Ecological, evolutionary & environmental sciences

For a reference copy of the document with all sections, see nature.com/documents/nr-reporting-summary-flat.pdf

Life sciences study design

All studies must disclose on these points even when the disclosure is negative.

Sample size	No sample size calculation was performed. This represents a retrospective multicenter study where all consecutive patients with matched samples of primary and recurrent BC were included (period 1999-2019). This represents one of the largest population analysed for this purpose so we believe our sample size is sufficient.
Data exclusions	Those patients for whom HER2 status evaluation was not available on both primary tumor and matched relapse samples were excluded. Patients experiencing contralateral breast cancer in the absence of other sites of recurrence were excluded as well.
Replication	N/A
Randomization	N/A
Blinding	N/A

Reporting for specific materials, systems and methods

We require information from authors about some types of materials, experimental systems and methods used in many studies. Here, indicate whether each material, system or method listed is relevant to your study. If you are not sure if a list item applies to your research, read the appropriate section before selecting a response.

Materials & experimental systems

n/a	Involved in the study	
☒	☐	Antibodies
☒	☐	Eukaryotic cell lines
☒	☐	Palaeontology and archaeology
☒	☐	Animals and other organisms
☐	☒	Human research participants
☐	☒	Clinical data
☒	☐	Dual use research of concern

Methods

n/a	Involved in the study	
☒	☐	ChIP-seq
☒	☐	Flow cytometry
☒	☐	MRI-based neuroimaging

Human research participants

Policy information about studies involving human research participants

Population characteristics

ALL RELEVANT POPULATION CHARACTERISTICS WERE INCLUDED IN THE MANUSCRIPT.

547 patients were retrosepctively included

Median age at diagnosis: 51.6yy.

Primary BC phenotype:
- HR+/HER2- 336 (61.4)
- triple-negative 79 (14.5)
- HER2+ 132 (24.1)

Treatment for early BC:
--->Adjuvant
- Chemotherapy 282 (51.5)
- Hormonal therapy 350 (64.0)
- Anti-HER2 68 (12.4)

-->Neoadjuvant
- Chemotherapy 151 (27.6)
- Hormonal therapy 14 (2.5)
- Anti-HER2 37 (6.8)

nature portfolio | reporting summary

	Treatment for relapsed/stage IV BC:<br>- Chemotherapy 430 (78.6)<br>- Hormonal therapy 364 (66.5)<br>- CDK 4/6 inhibitors 40 (7.3)<br>- Anti-HER2 131 (23.9)
Recruitment	This represents a retrospective multicenter study including all consecutive patients with matched samples of primary and recurrent BC from 1999 to 2019
Ethics oversight	Istituto Oncologico Veneto – IRCCS, Padova and Treviso Hospital, Italy Istitutional Review Boards.<br>This study has been performed in accordance with the Declaration of Helsinki. All the patients provided written-informed consent prior to inclusion into the study.

Note that full information on the approval of the study protocol must also be provided in the manuscript.

Clinical data

Policy information about clinical studies

All manuscripts should comply with the ICMJE guidelines for publication of clinical research and a completed CONSORT checklist must be included with all submissions.

Clinical trial registration	N/A (This is NOT a clinical trial)
Study protocol	This is NOT a clinical trial. However, documents related to the study protocol submitted and approved by the abovementioned ethical committees are available upon request.
Data collection	This is NOT a clinical trial<br>Retrospective data collection in a dedicated database after data anonymization
Outcomes	This is NOT a clinical trial.<br>Retrospective study: the primary objective was to evaluate the evolution of HER2-low expression from primary breast cancer to matched locoregional recurrences/distant metastases.
