# Supplementary material for: Evolution of HER2-low expression from primary to recurrent breast cancer
Source: NPJ Breast Cancer. 2021 Oct 12;7:137. doi: 10.1038/s41523-021-00343-4 (PMC8511010; doi:10.1038/s41523-021-00343-4)

Supplementary methods

The immunohistochemistry assays for HER2 adopted were (all FDA approved):

- Center #1
    - Until 2008 Hercept-Test®, DAKO (ref. number 2007333; 1:1000 dilution)
        - Number of primary BC cases=223*
        - Number of relapse biopsy cases=35
    - 2009-2011 Pathway®, Ventana (clone 4B5) (ref. number 780-4422; 1:3000 dilution)
        - Number of primary BC cases=131*
        - Number of relapse biopsy cases=74
    - Since 2012 Bond Oracle®, Leica (clone CB11) (ref. number 780-4422, 1:1000 dilution)
        - Number of primary BC cases=144*
        - Number of relapse biopsy cases=390

  *1 missing case

- Center #2
    - Until 2016 Hercept-Test®, DAKO (ref. number 2007333; 1:1000 dilution)
        - Number of primary BC cases=37
        - Number of relapse biopsy cases=5
    - Since 2016 Pathway®, Ventana (clone 4B5) (ref. number 780-4422; 1:3000 dilution)
        - Number of primary BC cases=11
        - Number of relapse biopsy cases=43

No differences in HER2 expression discordance was observed across different IHC protocols (p=0.177).

Supplementary results

	HR+/HER2 (N=336)				TN (N=79)			
	**HER2-0**	**HER2-LOW**	**p**	**Total**	**HER2-0**	**HER2-LOW**	**p**	**Total**
**Age at primary BC diagnosis, median yy (Q1-Q3)**	52.8 (43.2-61.8)	54.3 (45.5-65.4)	0.095	53.6 (44.2-63.0)	52.1 (41.3-61.1)	53.4 (44.6-64.7)	0.730	52.3 (42.5-61.2)

**Histotype, n (%)**	**Ductal**	125 (71.8)	131 (82.9)	0.056	256 (76.2)	43 (84.3)	24 (85.7)	0.986	67 (84.8)
	**Non-ductal**	49 (28.2)	27 (17.1)		76 (22.6)	8 (15.7)	4 (14.3)		12 (15.2)
	**Missing**	4 (1.2)				0 (0)			
**Histologic Grade, n (%)**	**1**	12 (7.3)	13 (8.5)	0.190	25 (7.4)	0 (0.0)	0 (0.0)	0.452	0 (0.0)
	**2**	88 (53.3)	66 (43.1)		154 (45.8)	4 (8.2)	4 (14.3)		8 (10.1)
	**3**	65 (39.4)	74 (48.4)		139 (41.4)	45 (91.8)	24 (85.7)		69 (87.3)
	**Missing**	18 (5.3)			18 (5.4)	2 (2.5)			
**Stage at diagnosis, n (%)**	**Early**	164 (93.7)	146 (93.0)	0.482	310 (92.3)	51 (100)	27 (96.4)	0.354	78 (98.7)
	**Stage IV**	11 (6.3)	11 (7.0)		22 (6.5)	0 (0.0)	1 (3.6)		1 (1.3)
	**Missing**	4 (1.2)				0 (0)			
**Primary BC Luminal-like phenotype, n (%)**	**Luminal A-like (Ki67 <20%)**	77 (48.1)	55 (35.2)	**0.023**	132 (39.3)	---	---	---	---
	**Luminal B-like (Ki67 >20%)**	83 (51.9)	101 (64.8)		184 (54.8)	---	---	---	---
	**Missing**	20 (6.0)				---			
**Primary BC ER, % mean (Q1-Q3)**		71.2 (60.0-90.0)	76.8 (70.0-90.0)	**0.032**	73.9 (70.0-90.0)	---	---	---	---
**Primary BC PgR, % mean (Q1-Q3)**		52.7 (20.0-80.0)	47.3 (13.75-80.0)	0.155	50.1 (15.0-80.0)	---	---	**---**	**---**
**Primary BCKi67, % mean (Q1-Q3)**		23.4 (10.0-34.3)	27.1 (15.0-38.7)	0.053	25.2 (10.0-35.0)	50.5 (25.0-70.0)	44.8 (28.5-67.5)	0.334	48.4 (28.0-70.0)

Supplementary Table 1 clinicopathological features of HER2-0 vs HER2-low BC according to primary BC subtype.

% are calculated among available case. % of total cases with missing information is also reported for each variable.

		**HR+/HER2 (N=301)**				**TN (N=116)**			
		**HER2-0**	**HER2-LOW**	**p**	**Total**	**HER2-0**	**HER2-LOW**	**p**	**Total**
**Age at primary BC diagnosis, median yy (Q1-Q3)**		55.8 (44.8-64.8)	50.3 (42.7-61.9)	**0.018**	53.4 (44.4-63.2)	52.3 (43.9-62.3)	52.1 (42.2-63.0)	0.945	52.2 (43.1-62.2)
**Recurrent BC Luminal-like phenotype, n (%)**	**Luminal A-like (Ki67 <20%)**	38 (40.9)	40 (36.4)	0.563	78 (25.9)	---	---	---	---
	**Luminal B-like (Ki67 >20%)**	55 (59.1)	70 (63.6)		125 (41.5)	---	---		---
	**Missing**	98 (32.6)				---			
**Recurrent BC ER, % mean (Q1-Q3)**		77.53 (70.0-95.0)	79.41 (80.0-90.0)	0.529	78.6 (70.0-90.0)	---	---	---	---
**Recurrent BC PgR, % mean (Q1-Q3)**		29.5 (0.0-62.5)	37.4 (0.0-70.0)	0.632	30.6 (0.0-70.0)	---	---	**---**	**---**
**Recurrent BC Ki67, % mean (Q1-Q3)**		25.4 (10.0-40.0)	27.9 (15.0-40.0)	0.350	26.7 (12.0-40.0)	38.9 (10.0-65.5)	44.2 (20.0-61.2)	0.434	40.9 (11.2-64.2)

Supplementary Table 2 clinicopathological features of HER2-0 vs HER2-low BC according to recurrent BC subtype.

		Primary BC			Recurrent/Stage IV BC		
		HER2-0	HER2-low	p	HER2-0	HER2-low	p
**Primary BC sample**	**Surgery**	174 (58.8)	122 (41.2)	0.016	-	-	-
	**Biopsy**	54 (45.4)	65 (54.6)		-	-	-
**Relapse samples**	**Locoregional relapse**	-	-	-	84 (50.3)	83 (49.7)	0.765
	**Distant metastasis**	-	-	-	131 (52.0)	121 (48.0)	
**Relapse sites**	**Liver**	-	-	-	61 (46.6)	70 (53.4)	0.443
	**Lung/pleura**	-	-	-	17 (51.5)	16 (48.5)	
	**Bone**	-	-	-	16 (66.7)	8 (33.3)	
	**CNS**	-	-	-	4 (50.0)	4 (50.0)	
	**Lymph-node**	-	-	-	38 (59.4)	26 (40.0)	
	**Soft tissue/skin**	-	-	-	67 (48.6)	71 (51.4)	
	**Other sites**	-	-	-	12 (57.1)	9 (42.9)	

Supplementary Table 3 HER2 expression according to type of tumor sample in the HER2-negative cohort

		HER2 discordance status		
		**HER2 concordant**	**HER2 discordant**	**p**
**Age at primary BC diagnosis, median yy (Q1-Q3)**		51.1 (43.2-63.4)	52.0 (43.7-62.1)	0.957
**Primary BC histotype, n (%)**	**Ductal**	274 (81.5)	163 (79.5)	0.273
	**Non-ductal**	62 (18.5)	43 (20.5)	
**Primary BC histologic Grade, n (%)**	**1**	14 (4.3)	13 (5.2)	0.328
	**2**	113 (34.7)	74 (37.8)	
	**3**	199 (61.0)	109 (55.6)	
**Stage at diagnosis, n (%)**	**Early**	315 (94.0)	194 (94.2)	0.552
	**Stage IV**	20 (6.0)	12 (5.8)	
**Primary BC phenotype, n (%)**	**HR+/HER2-**	183 (54.1)	153 (73.6)	**<0.001**
	**TN**	50 (14.8)	29 (13.9)	
	**HER2+**	105 (31.1)	26 (12.5)	
**Primary BCKi67, % mean (Q1-Q3)**		30.3 (15.0-40.0)	28.7 (10.0-40.0)	0.304

Supplementary Table 4 clinicopathologic features according to HER2 discordance status

Supplementary Figure 1 HER2 evolution from primary BC to relapse according to IHC scores

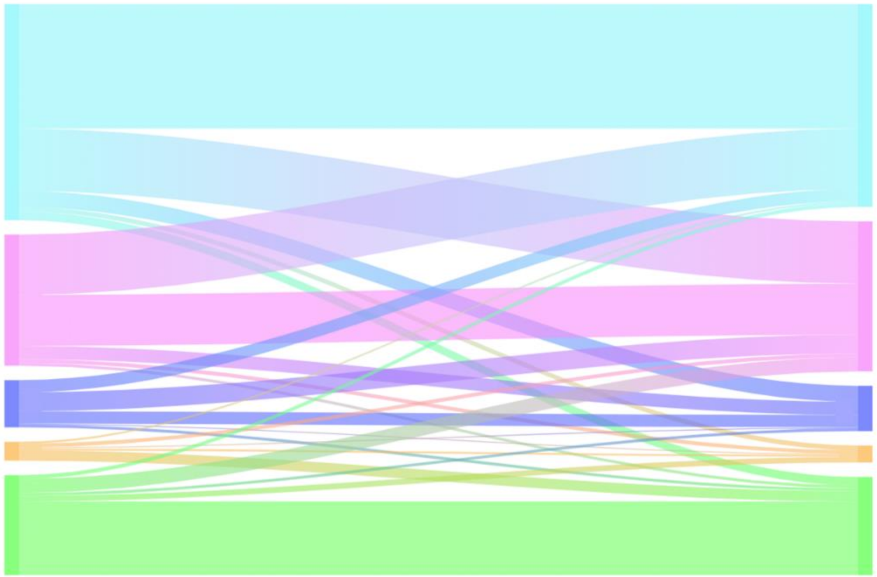

		HER2 recurrence/metastasis N,%				
		**0**	**1+**	**2+ N.A.**	**2+ A.**	**3+**
**HER2 primary BC N,%**	**0**	132 (24.3%)	66 (12.1%)	17 (3.1%)	5 (0.9%)	9 (1.7%)
	**1+**	64 (11.8%)	54 (9.9%)	14 (2.6%)	4 (0.7%)	3 (0.6%)
	**2+ N.A.**	13 (2.4%)	20 (3.7%)	13 (2.4%)	1 (0.2%)	3 (0.6%)
	**2+ A.**	2 (0.4%)	4 (0.7%)	1 (0.2%)	2 (0.4%)	11 (2%)
	**3+**	4 (0.7%)	15 (2.8%)	3 (0.6%)	6 (1.1%)	78 (14.3%)

(2 patients in the primary cohort and 6 patients in the recurrent cohort had IHC scores 0 or 1+ with *HER2* gene amplification by FISH)

Abbreviations; N.A., *HER2* not amplified by FISH; A., *HER2* amplified by FISH

Supplementary Figure 2 A) Kaplan Meier curves patients with HR-positive/HER2-negative phenotype in both primary and recurrent BC samples, according to HER2-low expression evolution from primary to recurrent BC B) Kaplan-Meier curves for PRS and C) OS in patients maintaining a HR-positive/HER2-negative BC phenotype from primary to recurrent BC, according to HER2-low expression on primary BC.

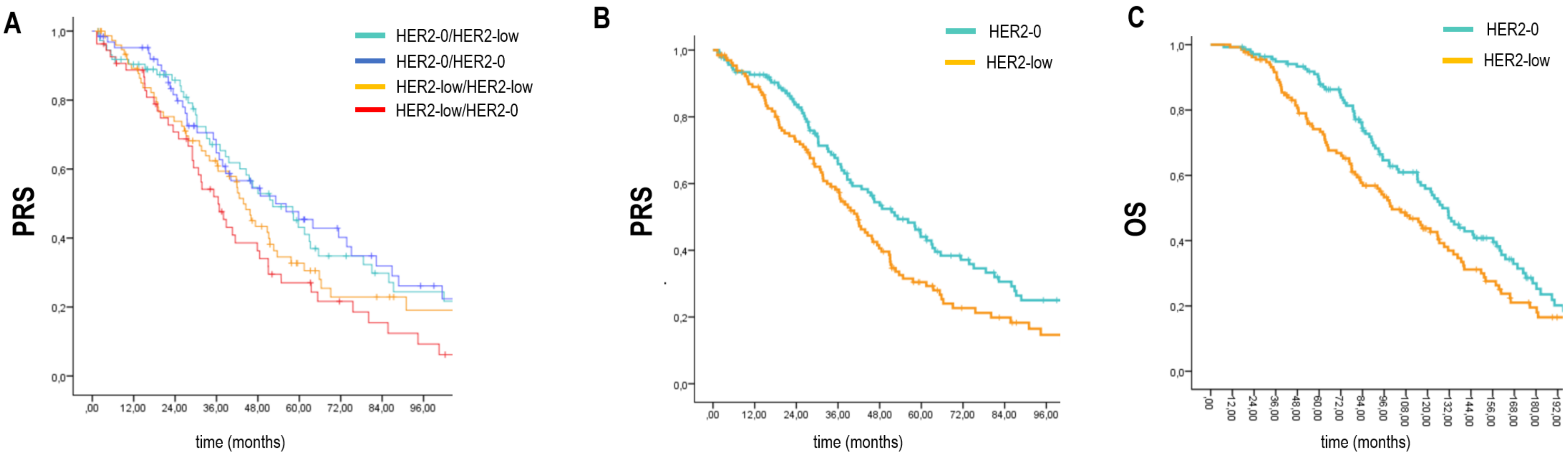

Supplement: Supplementary file 2 — Supplementary Information [file 41523_2021_343_MOESM2_ESM.pdf]
